# Spondyloarthritides: Theories and beyond

**DOI:** 10.3389/fped.2022.1074239

**Published:** 2022-12-23

**Authors:** Begüm Kocatürk, Zeynep Balık, Gaye Pişiren, Umut Kalyoncu, Füsun Özmen, Seza Özen

**Affiliations:** ^1^ Department of Basic Oncology, Hacettepe University Cancer Institute, Ankara, Turkey; ^2^ Division of Pediatric Rheumatology, Department of Pediatrics, Hacettepe University Faculty of Medicine, Ankara, Turkey; ^3^ Division of Rheumatology, Department of Internal Medicine, Hacettepe University Faculty of Medicine, Ankara, Turkey

**Keywords:** medical biology, spondyloarthritides, ankylosing spondylitis, enthesitis-related arthritis, rheumatology

## Abstract

Spondyloarthritides (SpA) are a family of interrelated rheumatic disorders with a typical disease onset ranging from childhood to middle age. If left untreated, they lead to a severe decrease in patients' quality of life. A succesfull treatment strategy starts with an accurate diagnosis which is achieved through careful analysis of medical symptoms. Classification criterias are used to this process and are updated on a regular basis. Although there is a lack of definite knowledge on the disease etiology of SpA, several studies have paved the way for understanding plausible risk factors and developing treatment strategies. The significant increase of HLA-B27 positivity in SpA patients makes it a strong candidate as a predisposing factor and several theories have been proposed to explain HLA-B27 driven disease progression. However, the presence of HLA-B27 negative patients underlines the presence of additional risk factors. The current treatment options for SpAs are Non-Steroidal Anti-Inflammatory Drugs (NSAIDs), TNF inhibitors (TNFis), Disease-Modifying Anti-Rheumatic Drugs (DMARDs) and physiotherapy yet there are ongoing clinical trials. Anti IL17 drugs and targeted synthetic DMARDs such as JAK inhibitors are also emerging as treatment alternatives. This review discusses the current diagnosis criteria, treatment options and gives an overview of the previous findings and theories to clarify the possible contributors to SpA pathogenesis with a focus on Ankylosing Spondylitis (AS) and enthesitis-related arthritis (ERA).

## Introduction

SpA is a group of immune system related disorders predominantly causing sterile inflammation at sacroiliac joints. In adults, patients often meet the definition of axial spondyloarthritis. In addition to AS, reactive arthritis, psoriatic arthritis, enteropathic arthritis, Reiter's syndrome, Inflammatory Bowel Disease (IBD)-associated arthritis and undifferentiated SpA can be included within this disease subset (1). The classification and terminology of juvenile SpA (JSpA) patients differ from the adults. Childhood onset patients are classified as ERA and juvenile onset psoriatic arthritis (JPsA) and juvenile idiopathic arthritis (JIA) is used as an umbrella term for these arthritides (2). In Europe and North America 10% of JIA patients are diagnosed as ERA (3, 4) whereas this ratio increases further to 35%–40% in Asia (5–8).

SpAs affects up to 2% of the population (1). The prevalence of the disease is highest in Europe followed by an Asian population whereas it is uncommon in Africans (9). SpA patients suffer from a significant decrease in their quality of life and may even need surgical operations as a remedy. The therapeutic agents used for the disease may cause side-effects (i.e., infection) and a certain portion of patients fail to respond to therapy (10, 11). Overall, it is clear that the development of alternative treatment strategies are necessary however, the obscure disease etiology plays a negative role in this process. Although there are studies underlining the possible contribution of HLA-B27 allele in disease pathogenesis, the presence of HLA-B27 negative SpA patients indicates the presence of extra risk factors (12).

## Clinical features

Similar to other diseases the early diagnosis of SpA is crucial. Delays may result in increased disease activity, irreversible structural damage, low therapy response and limited mobility (13). Physicians and patients should work hand in hand for early diagnosis to eliminate undesirable long-term effects. Diagnosis might be stalled if patients delay visiting a doctor due to limited access or in the belief that their symptoms will disappear spontaneously. Moreover, seeing other specialists rather than a rheumatologist might not only cause a delay but also may result in misdiagnosis (14).

**Figure 1:**
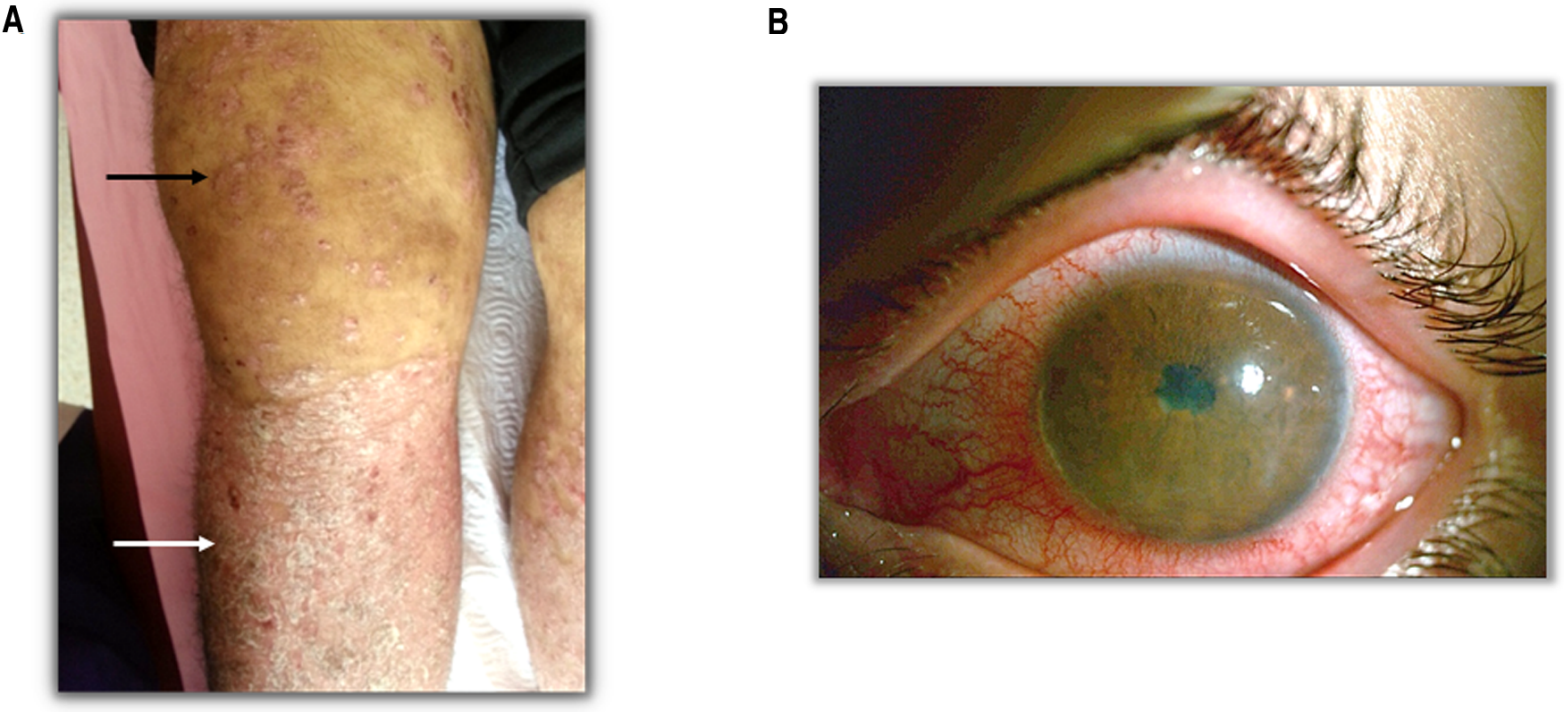
Clinical findings in SpA patients. (**A**) Black arrow shows the arthritis and white arrow shows the psoriasis. (**B**) A patient with anterior uveitis.

Thorough and distinctive analysis of the symptoms plays a fundamental role in the validity of diagnosis. SpA patients display several common clinical and laboratory findings such as arthritis (Figure 1A), psoriasis (Figure 1A), enthesitis, anterior uveitis (Figure 1B), inflammatory low back pain and family history of HLA-B27-related disease. Although the common features remain the same, the clinical phenotype differs across the ages in certain aspects with peripheral arthritis being predominant in JSpA and axial manifestations being more common in adult-onset disease (15). Inflammatory back pain is the most common complaint of SpA suggesting axial involvement. Shoulder and hip joint involvement is also more common in ERA (16). In fact the differences between childhood and adult- onset disease have been highlighted in a number of studies. Both are more common in males. Childhood cases typically present in adolescent years with arthritis in the big joints and often enthesitis (17). The most frequently involved joints are the knee (40%–50%), hip (30%–40%) and ankle (25%–40%) (16, 18). Axial disease and back pain are less than expected in adult-onset disease. The frequency of axial involvement differs between studies. In a systematic review of the literature, comparing juvenile-onset AS (JoAS) and adult-onset AS (AoAS) cohorts showed that axial disease is significantly more frequent in AoAS than JoAS cases (4.3%–74% vs. 56%–95%) (19). Again family history seems to be more common in childhood-onset disease and may suggest a higher genetic load associated with the disease. Despite these differences, the pathogenesis of these two different onsets are similar; thanks to the recent emerging data we know the main pathways involved and it may not be appropriate to classify the childhood-onset disease separately from the adult one, under the “idiopathic” term anymore.

In recent years MRI has improved the assessment and diagnosis of axial disease. Axial disease was probably underestimated before the widespread use of MRI both in children and adults. Approximately half of the patients are known to be first been diagnosed with sacroiliitis on MRI (20).

## Classification criteria

Most juvenile SpA are classified as enthesitis-related arthritis or undifferentiated arthritis, depending on whether psoriasis is present in the patient or their family. International League of Associations for Rheumatology (ILAR) criteria are used for the classification of JIA subtypes including ERA (Supplementary Table S1) (2, 21). According to the ILAR, ERA classification criteria is arthritis plus enthesitis or arthritis or enthesitis plus two of the following: (1) Sacroiliac joint tenderness and/or inflammatory back pain, (2) HLA-B27 positivity, (3) >6 years old boy and (4) Acute anterior uveitis and (5) Family history in at least one first degree relative of HLA-B27 associated disease like ankylosing spondylitis, ERA, sacroiliitis with IBD, reactive arthritis or acute anterior uveitis.

**Figure 2:**
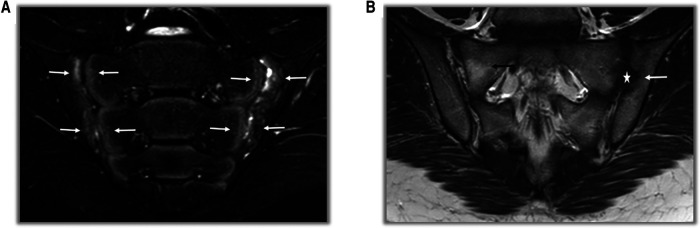
MRI findings in SpA patients. (**A**) On T2-weighted fat-suppressed coronal sections, areas of bone marrow edema consistent with acute sacroiliitis are observed on the facing sides of the bilateral sacroiliac joint (white arrows). (**B**) On T1-weighted fat-free coronal sections, erosion and irregularities at the level of the left sacroiliac joint (white arrow) and pseudo-widening (star) of the joint space are observed. In the right sacroiliac joint, there is an appearance of fat replacement (black arrow) in the sacral region.

In adults the diagnosis is based on the Assessment of SpA International Society (ASAS) classification (Supplementary Table S1). ASAS criteria include both imaging and clinical findings: if sacroiliitis is present on imaging [by radiographs or magnetic resonance imaging (MRI)] (Figure 2) only one other SpA feature is sufficent for classification. However, if imaging evidence of sacroiliitis is absent, positive HLA-B27 along with at least two other SpA features is required for the patient to be classified as having axial SpA. ASAS criteria for peripheral spondyloarthritis include peripheral arthritis and/or enthesitis and/or dactylitis plus 1 SpA feature (uveitis, psoriasis, Crohn's/colitis, preceding infection, HLA-B27, sacroiliitis on imaging) or ≥2 other SpA features (arthritis, enthesitis, dactylitis, inflammatory back pain, family history of SpA).

The ASAS and ILAR criteria set indeed overlap in the defined features and they share several clinical and laboratory findings for classification. However, there are also important differences between the two. Firstly, the ILAR system does not specifically address children who have SpA by fulfilling the criteria for ankylosing spondylitis, or who have coexisting conditions such as inflammatory bowel disease (22). Reactive arthritis, IBD-related arthritis, and psoriatic arthritis are not among the diagnostic criteria in ERA. While psoriatic arthritis in children is a different subgroup of JIA, both psoriatic arthritis, reactive arthritis, and IBD are included in the SpA group in adults (23). Secondly, the ILAR classification criteria focus on the importance of extra-axial manifestations such as peripheral arthritis and enthesitis, while SpA classification pays attention to the presence of axial and spinal involvement. Finally, elevated inflammatory markers is one of the criteria in SpA, whereas that is not the case in ERA.

Indeed the ILAR criteria has important limitations regarding the classification of patients in the spondyloarthropathy group. Enthesitis-related JIA was considered an undifferentiated SpA in ILAR, whereas all the different forms of adult SpA can be found in children, with the major difference being the higher proportion of undifferentiated forms in children. Thus a new classification criteria have been proposed by researchers from PRINTO, suggesting fundamental changes, in the classification of enthesitis-associated arthritis (24). This new criteria was called “Enthesitis/spondylitis-related JIA and included the following criteria: peripheral arthritis and enthesitis, or arthritis or enthesitis, plus ≥3 months of inflammatory back pain and sacroiliitis on imaging, or arthritis or enthesitis plus 2 of the following: (1) sacroiliac joint tenderness; (2) inflammatory back pain; (3) presence of HLA-B27 antigen; (4) acute (symptomatic) anterior uveitis; and (5) history of a SpA in a first-degree relative. Of note, if peripheral arthritis is present, it should persist for at least 6 weeks.

The current PRINTO definition has been partly harmonized with the adult one, and an imaging criterion for radiographs (25) or magnetic resonance imaging (26) has been introduced. The adult definition of inflammatory back pain has been adopted. Because the term ERA could wrongly suggest the existence of a form of SpA that is specific to childhood, it was initially proposed to name this condition juvenile SpA and was later changed to enthesitis/spondylitis-related JIA. Furthermore one of the main differences of PRINTO classification criteria is that sacroiliitis on imaging was added among the list. Albeit definition of sacroiliitis on MRI for adult patients well-defined, the use of this definition of MRI findings for pediatric cases may cause false-positive results due to the physiologic bone marrow changes of growing bone. Recently, MRI definitions for active and structural sacroiliac joint lesions in juvenile cases are published (27).

Although there is a lack of substantial molecular omics studies, most pediatricians would regard ERA more like a juvenile AS, especially once sacroiliitis is detected. There is a need for more follow-up data on patients with peripheral disease -fulfilling the ERA criteria, to understand whether they constitute a separate pediatric group. Moreover, sophisticated studies are crucial to understand whether axial-ERA is truly an early onset AS or SpA.

## Etiology

The lack of knowledge on SpA etiology has been a major concern in diagnosis and disease treatment. The diagnosis is dependent on clinical manifestations which shows heterogeneity between patients whereas the therapeutic interventions were developed based on observational studies. In this section we will discuss the possible risk factors for SpAs and theories related with them.

### HLA-B27

The immune system acts as a safeguard to protect our body from the invasion of harmful intruders. These foreign entities' proteins should be presented as peptides to our immune cells to activate a potent immune response. Peptides loaded on Major histocompatibility complex (MHC) molecules located on cell surface can be recognized by T cells which in turn cause their activation. All nucleated cells have MHC class I molecules that take part in the presentation of intracellular antigens (i.e., viral, tumor) to CD8+ T lymphocytes and their heavy chains are encoded by genes at HLA-A, HLA-B and HLA-C loci (28). Antigen presenting cells (APCs) play a significant role in activating the adaptive immune system and are specialized cells. MHC class II molecules are expressed on these cells and are involved in the presentation of exogenous peptides (i.e., bacteria, parasites) to CD4+ T cells. These molecules are encoded by HLA-DR, HLA-DP and HLA-DQ (29).

The first report showing the association of the MHC class I molecule HLA-B27 with SpAs was published in 1973 (30). Since then many studies were conducted to obtain more information on disease etiology and underlying mechanisms. HLA-B27 positive population constitutes 6%–8% of the general population whereas this ratio increases to more than 80% in AS patients (31, 32) implementing its strong plausible contribution to disease etiology. HLA-B27 has different variants with aminoacid substitutions mostly in their peptide binding cleft (33). Among these variants, HLA-B*27:05, HLA-B*27:02 and HLA-B*27:04 show association with SpA whereas this is not the case for HLA-B*27:06 and HLA-B*27:09 (34, 35). HLA-B*27:05 is more common in Caucasian, HLA-B*27:04 in Chinese and HLA-B*27:02 in Mediterranean population (36).

The importance of HLA-B27 in SpA etiology was also recapitulated using animal models. Rats having high levels of HLA-B27*05 and human beta 2 microglobulin (B27-Tg) partially phenocopy the human disease with inflammatory bowel condition, inflammatory peripheral arthritis and skin lesions (37). Interestingly, genetic factors seems to play a role in the process based on the fact that SpA related symptoms are only manifested in rats having Lewis or Fischer background but not in Dark Agouti background. Mice with same genetic modifications also display spontaneous arthritis (38) and the lack of β2-microglobulin (β2m) or TAP1 gene does not impair the manifestation of disease related phenotype (39, 40). The background of the mice has been found to be relative for the development of the disease as well (41).

The HLA-B27 levels seems to be a pivotial factor regulating disease susceptibility. Higher levels of HLA-B27 are typically seen in the peripheral blood mononuclear cells (PBMCs) of patients compared to healthy controls positive for this allele (42). Moreover, individuals homozygote for HLA-B27 are associated with an increased risk of AS development compared to heterozygotes (43). The same phenomenon is also observed in animal models. Disease susceptibility shows a positive correlation with HLA-B27 copy number and its relative expression in lymphoid cells (44) that can be upregulated *via* pro-inflammatory stimuli. Of note, this dose dependent effect might also explain why only 2% of HLA-B27 positive patients develop the disease.

The positivity of HLA-B27 also has an influence on disease manifestation. In more than 80% of AS patients, symptoms emerge at ≤30 years of age. Interestingly, HLA-B27 positive AS patients show an earlier disease onset compared to negative ones (45) and have a worse prognosis with elevated disease activity and duration (46). The frequency of specific symptoms also depends on HLA-B27 status. Psoriasis and IBD are more common in HLA-B27 negative patients whereas peripheral arthiritis and uveitis are observed more frequently in HLA-B27 positive ones (47).

Overall, it is clear that HLA-B27 somehow plays a role in disease pathogenesis. Its possible contribution to disease progression and related theories are discussed below.

#### Arthritogenic peptide/molecular mimicry hypothesis

The mature MHC I molecule is composed of a heavy chain (HC), a β2m light chain and a peptide, 8–10 amino acids in length. Its formation involves a series of protein assembly and disassembly within the complex. First, newly synthesized heavys chains are translocated to the endoplasmic reticulum (ER) and glycosylated. This post-translational modification acts as a signal for incomplete folding which in turn triggers HCs interactions with chaperones calnexin and calreticulin. As HCs gain the correct tertiary structure, they associate with β2m resulting in the dissociation of calnexin (48, 49). Next, the complex further interacts with a transporter associated with antigen processing (TAP) *via* tapasin which is bound to ERp57 to form the peptide loading complex (48). Eventually, Erp57 and calreticulin dissociate to allow the binding of peptides to the MHC I. Although, MHC class I molecules are responsible for the presentation of the peptides to CD8+ T cells, these peptides should be trimmed before they are loaded on the complex. For that, purpose proteasome performs the initial trimming process causing the formation of peptides ∼15 aa in length. These peptides enter ER through TAP transporter and furher cleaved by ERAP1 and ERAP2 to have the optimal length for the loading (50). Finally, as MHC I is loaded with the peptide, the complex is sent to the surface of nucleated cells in particular APCs to perform a successful round of peptide presentation.

The plausible contribution of APCs in disease pathogenesis has been the center of many studies. The increased abundance of macrophages in sacroiliac (51) and enthesis (52) biopsies of AS patients attain a possible role for these cells. The level of circulating CD141+ dendritic cells (DCs) show a positive correlation with BASDAI in AS patients (53). Moreover, lower levels of MHC class II expression in DCs of AS patients (54) and animal model (55) implies that distortions in antigen presentation might very well be a key factor in disease pathogenesis.

**Figure 3:**
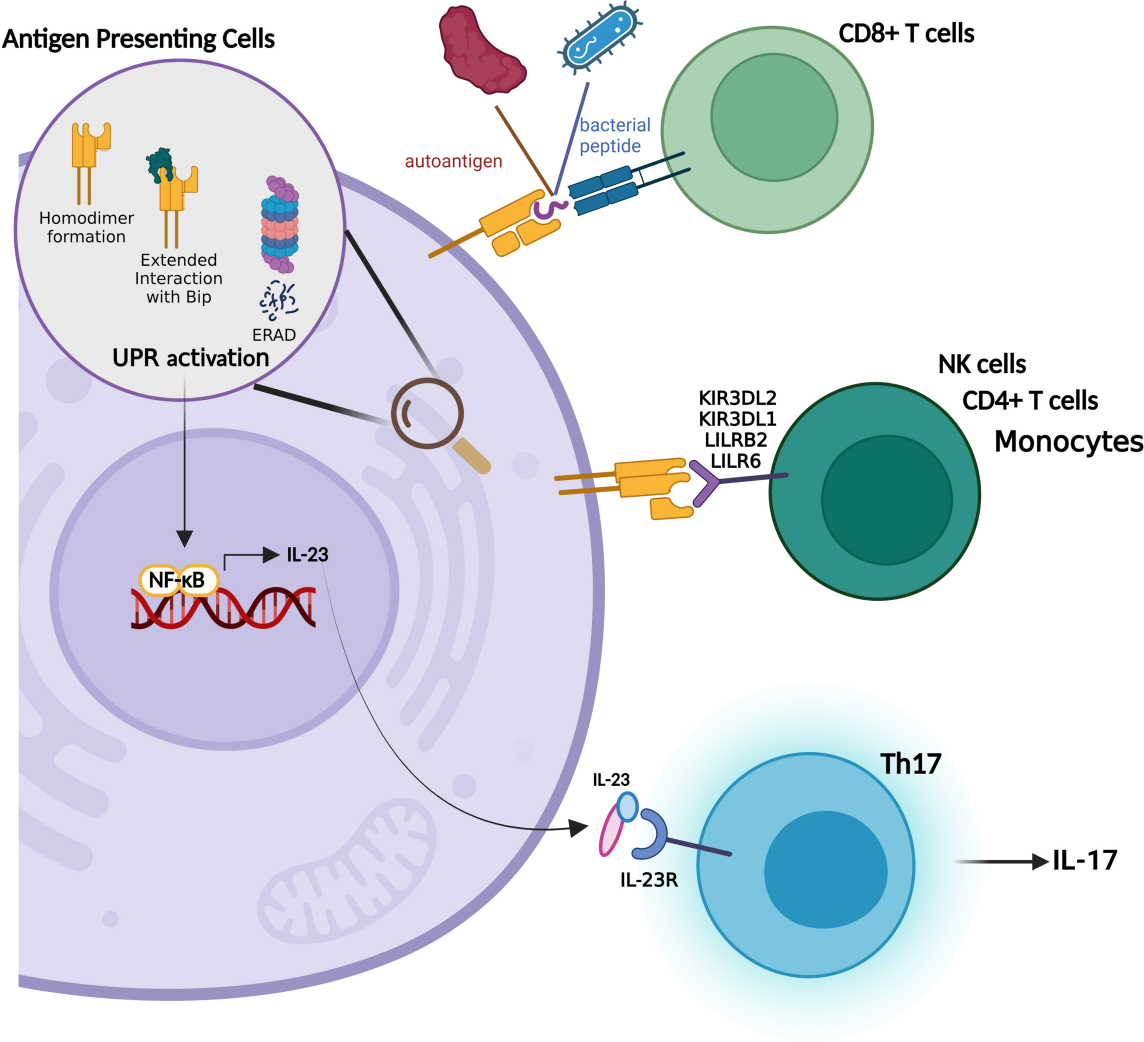
HLA-B27 related theories in SpA pathogenesis.

Previous studies suggested that HLA-B27 binds to a distinctive set of peptides that show similarity to self-peptides (Figure 3). Their presentation to CD8+ T lymphocytes triggers the breakdown of self tolerance which in turn activates a destructive immune response in affected sites (56). In support of this notion, HLA-B27-restricted CD8+ T cells were detected in the synovial fluid of AS patients (57, 58). They are also found to be directed against self-peptides derived from vasoactive intestinal peptide type 1 receptor (VIP1R, aminoacids 400–408) and glucagon receptor (GR, aminoacids 412–420) (59, 60). A controversial finding pointed out that HLA-B*27:09 subtype that is not associated with the disease also presents the VIP1R-derived peptide (61). However, further investigations revealed that peptide's conformation differs from the one presented by the disease relevant variant HLA-B*27:05 (59).

The molecular mimicry between HLA-B27 and gram-negative bacteria was also suggested to be a key element in autoreactive T cell activation and autoimmune reaction. Indeed, the sequence homology between HLA-B27 and arthritogenic bacterias *Klebsiella pneumoniae* (62), *Yersinia enterocolitica*, *Salmonella typhimurium*, *Shigella flexneri* and *Shigella sonnei* was described (63). This theory is further supported by a study by Ramos et al. showing that a peptide derived from the intracytoplasmic tail of HLA-B27 shows similarity to *Chlamydia trachomatis* and acts as a ligand only for disease associated HLA-B27 variants (64).

Although presentation of unusual peptides by HLA-B27 was suggested in disease pathogenesis, the ongoing presentation of disease related symptoms in CD8+ T cell depleted B27-Tg rats or TAP1^−/−^ mice argues strongly against the importance of antigen presentation in disease etiology (65, 66) thus other theories were also developed (40, 65, 66).

#### Homodimeric HLA-B27 molecule

As mentioned above, the major function of a MHC class I molecule which is composed of a heavy chain, β2m and a peptide, is to present these peptides to CD8+ T cells. Rather, HLA-B27 was reported to be recognized by Natural Killer Cells (NKs) and CD4+ T lymphocytes in the form of β2m-free homodimers (67) which is established through an unpaired cysteine at position 67 (68) (Figure 3). Strikingly, the B27-Tg rats with functional NK cells do not present disease symptoms indicating that these cells act in concert with lymphoctes in disease manifestation (69). As a matter of fact the critical involvement of CD4+ T cells in disease progression was recapitulated in many studies. Transfer of different T lymphocyte populations to athymic nude B27-Tg mice revealed that CD4+ T cells are the major cell population inducing colitis (70). Moreover, their levels shows an increment in the lymph nodes draining the sites of inflammation in animal model (71) and in peripheral blood of SpA patients (72). Higher levels of activated CD4+ T cells is also observed in B27-Tg rats compared to healthy ones (65).

Studies aiming to understand the involvement of dimer formation in disease pathogenesis unearthed that HLA-B27 variants associated with SpA have an increased tendency for dimer formation (73). The receptors for HLA-B27 homodimers were found to be KIR3DL1, LILRB2 (74), KIR3DL2 (75) and LIR6 (68) and expressed on lymphocytes, monocytes and Natural Killer Cells (NKs) (75). The level of KIR3DL2 positive NK and CD4+ T cells increases in the peripheral blood and synovial fluid of SpA and ERA patients. The receptor engagement also shows a survival and activation profile in NKs and T cells respectively (73, 76) whereas the dissociation between KIR3DL2 and HLA-B27 decreases the survival/proliferation of NKs and the release of disease related cytokine IL17 from the PBMCs of AS patients (77). In line with this finding, KIR3DL2+ CD4+ T cells collected from the synovial fluid of SpA patients displays enhanced levels of IL17 secretion (78).

#### Unfolded protein response (UPR) activation

UPR activation was suggested to be a major explanation in SpA pathogenesis. Proteins are biomolecules that orchestrate many cellular processes. To fulfill their task, they need to be folded properly in the organelle called Endoplasmic Reticulum (ER). Proteins with folding deficits can be removed *via* autophagy or Endoplasmic Reticulum Associated Degradation (ERAD) (79). However, the accumulation of misfolded proteins may also take place which in turn activates a stress response machinery namely UPR. This stress is regulated through 3 transmembrane proteins located on ER: Inositol-requiring enzyme 1 (IRE1), PKR-like ER kinase (PERK) and Activating Transcription Factor 6 (ATF6). Upon its activation, IRE1 cleaves Xbp mRNA leading to the formation of an active transcription factor sXbp. This factor is responsible for the synthesis of chaperones and ERAD components to achieve cellular homeostasis whereas an increase in magnitude and duration of stress results in the activation of the apoptotic IRE1-JNK pathway. Activation of PERK leads to the phosphorylation of eIF2α which in turn put a halt to translation whereas activating selective translation of the ATF4 transcription factor. Similar to IRE1 pathway, ATF4 is also responsible for the activation of homeostatic genes yet unresolved stress promotes the expression of pro-apoptotic CHOP. ATF6 is translocated to Golgi upon UPR and cleaved with S1P and S2P proteases. The newly formed cytosolic domain of ATF6 is a transcription factor and is involved in the transcription of chaperones (80).

HLA-B27 is unique in a way that it misfolds even in the presence of β2m and peptides which in turn activate UPR (Figure 3). There are several bodies of evidence proving this aberrant feature of HLA-B27:

- Folding rate: HLA-B27 has a slow folding nature which in turn induces homodimer formation and its retention in the ER. These molecules can then activate the UPR. The B pocket which is located at the peptide binding groove of HLA-B27 seems to be crucial for this machinery. Altering residues in this region not only enhances HLA-B27's folding but also alleviates homodimer formation (81).
- ERAD: Another clue showing the misfolded nature of HLA-B27 is its enhanced predisposition to undergo ERAD (82) in which EDEM1, and HRD1 were found to be pivotal regulators (83, 84). In support of this notion, the use of ERAD blocking reagents results in an increment in the levels of HLA-B27 dimers/oligomers.
- Interaction with chaperones: The chaperones help misfolded proteins to gain a proper tertiary structure. The prolonged interaction between HLA-B27 multimers and chaperone Bip indicates the improper folding of HLA-B27 which in turn activates the stress response (85). In addition, the enhanced interaction between HLA-B27 and oxidoreductase Erp57 is also involved in dimer formation which again may turn on UPR (86).

Macrophages residing in the peripheral joints of AS patients have increased levels of Bip compared to osteoarthritis patients (87) and mononuclear cells collected from the synovial fluid of SpA patients shows an activation state for UPR (88). In B27-Tg rats, bone marrow derived macrophages shows prominent UPR activation status which shows a positive correlation with HLA-B27 levels (89, 90). The UPR induction was also observed in B27-Tg rats' dendritic cells (55). Strikingly, ERAP1 deficient B27-Tg rats remained healthy due to the blockade of UPR activation (91).

The pathogenesis of SpA clearly involves the activation of the immune system thus UPR-driven immune modulation has also been the subject of extensive investigation. NF*κ*B was shown to be activated during UPR (92) that mediates Th17 differentiation *via* IL23, a cytokine that is elevated in the serum and synovial fluid of SpA patients (93, 94). The activated Th17 cells in turn produce cytokines such as IL17, TNF and IL6 (95). A strong activation for IL23/IL17 axis was detected in the colon of B27-Tg rats (96) and overexpression of IL23 causes a disease phenotype similar to AS in mice (97). In addition, Th17 cells were found to be enriched in the peripheral blood of AS patients (98). Furthermore, DCs, a major source for IL23, contribute to Th17 cells' expansion in the B27-Tg animal model (71). Although, macrophages with prominent UPR activation are destined to produce higher levels of IL23 (96), there are also studies showing that IL23 production is independent of UPR activation thus further studies are warranted (99).

Another NF*κ*B dependent cytokine TNF-α is also a critical component of the disease and also used as a target for therapy. Similar to IL23, overexpression of TNF results in spondyloarthritis formation in mice and this process was found to be regulated through mesenchymal cells (100). The level of Bip in the macrophages collected from the synovial fluid shows a positive correlation with TNF levels indicating that immune modulation by UPR might be the basis for elevated TNF levels in disease (87). TNF is detected in the inflamed tissues of SpA patients and also is elevated in PBMCs and serum (101).

### Other susceptibility genes

Studies on families revealed that SpAs may have a heritable component. For JIA the recurrence risk in first cousins was determined to be 5.8 fold whereas the sibling relative risk is estimated to be 11.6 fold (102). AS's heritability is ≥90% (103, 104) with a sibling recurrence risk of 8.2% (105). The prevalence increases dramatically with the presence of a first degree relative suffering from the disease (106, 107) and the concordance rate was determined to be 25%–75% and 4%–15% in monozygotic and dizygotic twins respectively (103, 104, 108). Overall, these findings strongly indicate that genetic factors are key determinants in disease pathogenesis. As mentioned above the presence of HLA-B27 showed the highest association with disease susceptibility. However, the fact that only 2% of the HLA-B27 positive population develops SpA is a strong indicator that there are additional genetic risk factors for the development of the disease (109). To understand this phenomenon better genome wide association studies (GWAS) were performed in SpA patients.

The second well established susceptibility locus for AS and ERA was found to be ERAP1 (110, 111) which is involved in the presentation of peptides with optimal length. Several studies made it apparent that defects in ERAP function might be involved in disease pathogenesis. ERAP1 variants with a loss of function shows a protective effect for disease which provides strong evidence for the involvement of atypical processing of antigenic peptides (112). Indeed, HLA-B27 was shown to bind extended peptides with protruding C-terminus (113) which in turn may activate a potent T cell response thus leading to SpA development. The deficits in peptide trimming might also decrease the level of peptide- loaded MHC molecules which in turn increase the levels of misfolded HLA-B27 molecules, UPR activation and disease progression. Elevated UPR levels might also be regulated through other mechanisms including damaged ubiquitin-ERAD machinery. Previous studies clearly show that ubiquitin conjugating enzyme UBE2J1 is involved in targeting of MHC class I molecules for ERAD (83) thus further studies aiming to unveil the link between another susceptibility gene UBE2E3 (114) and ERAD in disease etiology would be valuable.

Shaping immune response is indispensable in AS pathogenesis. As mentioned above, the IL23/IL17 axis is a crucial component of this machinery. Its importance was also verified in GWAS studies. Molecules related with this pathway (IL23R, IL12B, IL6R, IL1R1, IL1R2, TYK2, IL27A, STAT3, JAK2) are among the gene loci that shows association with AS (83, 114). This also holds true for the TNF-α pathway. Research revealed the presence of disease associated SNPs near to/in TNFRSF1A (83, 114), TNFSF15 (115) and TRADD (116). Genes related to T cell regulation, RUNX3, IL7R, EOMES, ZMIZ1, ICOSLG, SH2B3 and BACH2, are also among the AS risk loci (9). Other genes showing association with AS are GPR25, GPR65, GPR35, TBKBP1, PTGER4, BACH2, NOS2, FCGR2A, NKX2-3 (9), CARD9 (117, 118), KIF21B (119), ANTXR2 (120), ANO6 (121).

For JIA, ERAP1and IL23R are among the disease susceptibility genes for ERA and juvenile psoriatic arthritis respectively (111). The lower prevalence of JIA subtypes hampers the construction of well-powered cohorts for GWAS analysis. Thus combining all JIA subtypes rather than investigating them separately was used to detect genetic associations. PTPN2, ANGPT1, COG6 (122), CD80, JMJD1C (123), TRAF1-C5 (124), VTCN1 (125), IL2RA, IL2RB, STAT4 (126), TNFAIP3 and TRAF1/C5 (127) were found to be JIA-predisposing loci.

### Gender

Many rheumatic diseases display gender predominance. Both the incidence of AS (109) and ERA (128) are higher in males. However, the male to female ratio has showed decrement over time (129). This gender predominance indicates that sex-specific factors might play a role in SpA pathogenesis. Among these factors the impact of hormones in SpA progression was extensively analyzed. Of note, the age interval for ERA patients is 6–16 and it is well known that the level of sex hormones increases with puberty thus the following observations may partially explain the male dominance of ERA.

The effect of TNF inhibitors on SpA progression underlines TNF's importance in disease progression. Interestingly, estrogen was shown to decrease inflammation in SpA patients *via* downregulating TNF alpha levels (130). Estrogen supplementation was also shown to decrease disease severity both in human (131) and animal female subjects (132) whereas there are other studies showing no evident association (133). Testosterone levels on the other hand did not show any difference between SpA patients and healthy controls and were not likely to regulate disease progression (131, 134). Sex hormones were also shown to regulate the microbiome (135) and immune system (136). The fact that both of these factors play a role in disease progression (see below) underlines the presence of a possible hormone-driven microbiome and immune system related axis however, further studies are needed.

### The gut and microbiome

The gut is one of the affected sites in SpA. Inflammatory bowel disease (Crohn's disease and ulcerative colitis) occur concomitantly in up to 10% of SpA positive population (137). Patients display inflammatory lesions at intestinal mucosa (138) and the gut is also inflamed in the animal model (139) indicating that an active immune response in the gut and SpA might be interlinked. In support of this notion, macrophages expressing the CD163 scavenger receptor increases in the colonic mucosa of SpA patients (140) and IL23/IL17 axis is exacerbated in the colon of B27-Tg rats (96).

The relation between SpA development and microbiota has been the center of attention for decades. In animal models, housing of the animals in pathogen-free conditions alleviated the formation of several disease related symptoms including colitis and arthritis. However, their transfer to conventional conditions caused their manifestation. Moreover, treating B27-Tg rats with antibiotics hampered colitis formation (141) indicating that the microbiome is a key player in disease pathogenesis (39, 142). Indeed, there are studies showing the differences in microbiome of SpA patients and healthy controls (143–145) and ileal biopsies from AS patients revealed the presence of adherent and invasive bacteria which is accompanied by the decreased barrier function of the gut (146). Mucins play a major role in barrier function. Mucin-degrading *Akkermansia muciniphila* species was found to be elevated in B27-Tg rats indicating that SpA related dysbiosis may be involved in impaired gut barrier (147). T cells are another key player for maintaining the tolerance against commensal bacteria (148). Interestingly, CD4+ T cells isolated from B27-Tg rats produces higher levels of IFN-γ in response to antigens derived from these organisms implying that there might be a loss of tolerance for the microbiome (149, 150). Moreover, the defective stimulation of T cells by APCs might also contribute to the loss of tolerance for microbial flora (151, 152).

The link between treatment response and microbiome was also investigated. Patients receiving 3 months of anti-TNF therapy did not show a significant difference in their microbiata composition. However, having higher levels of *Burkholderiales* prior to therapy and an increment in genus Dialister after therapy was observed in responders (153).

### Diet

Diet plays a crucial role in the development and progression of many diseases. Diet has also been investigated in SpA however, most studies were not replicated. A study by Haugen et al. indicated that many AS patients reported that diet plays a role in the manifestation and severity of their symptoms (154) and they follow certain diets to decrease their intensity (155).

Starch consumption was suggested to be an exacerbating factor in SpAs and a low starch diet was found to lower disease activity whereas there are also studies showing no impact (155, 156). Salt and dietary fat consumption did not show any correlation with the severity of the symptoms (155, 157). Although quitting dairy products seem to have an ameliorating role in disease (158) there are also studies showing no effect (157).

In human subjects, the impact of prebiotic uptake in SpA progression was analyzed. SpA patients with concomitant quiescent ulcerative colitis receiving *Lactobacillus acidophilus* and *Lactobacillus salivarius* displayed lower disease activity (159). In contrast, a meta-analysis by Sanchez et al. opposed this finding (160). In animal model the severity of colitis was diminished with the supplementation of diet with prebiotics (161). The constituent, fructo-oligosaccharides was found to have the greatest anti-inflammatory effect in this regard (162, 163). Fibre-rich diets also showed a remedial effect on disease by upregulating short chain fatty acids. Indeed, administration of propionate to B27-Tg animals attenuates intestinal inflammation (164).

## Treatment and outcome

There are several treatment options used in clinics for SpAs. However, the current therapy options do not always result in full remission. Treatment of ERA varies according to whether the disease is axial or peripheral, the number of active joints, the presence of risk factors, and accompanying extra-articular features (Table 1). NSAIDs are used as the first-line treatment in enthesitis and sacroiliitis because of their analgesic and anti-inflammatory effects. For peripheral disease, DMARDs, especially methotrexate or salazopyrin are recommended. Sulfasalazine or methotrexate is used for enthesitis or active peripheral arthritis (165). The response to these non-biologic DMARDs varies in a wide range (166). Non-biologic DMARDs can also be used to prevent the development of anti-drug monoclonal antibodies against TNF inhibitors (TNFis) (167). Methotrexate and Salazopyrin monotherapy is not recommended in active sacroiliitis whereas they can be used as an adjunct therapy. If arthritis does not respond to non-biologic DMARDs or for patients who develop the axial disease then biologic DMARDs would be indicated, often along with the NSAID treatment. Among these, anti-TNF drugs are the first choice. Since etanercept and adalimumab are licensed for pediatric use, the present data is mainly focused on the effectiveness and safety of these two monoclonal anti-TNF drugs (168, 169).

**Table 1:** ERA treatment algorithm.

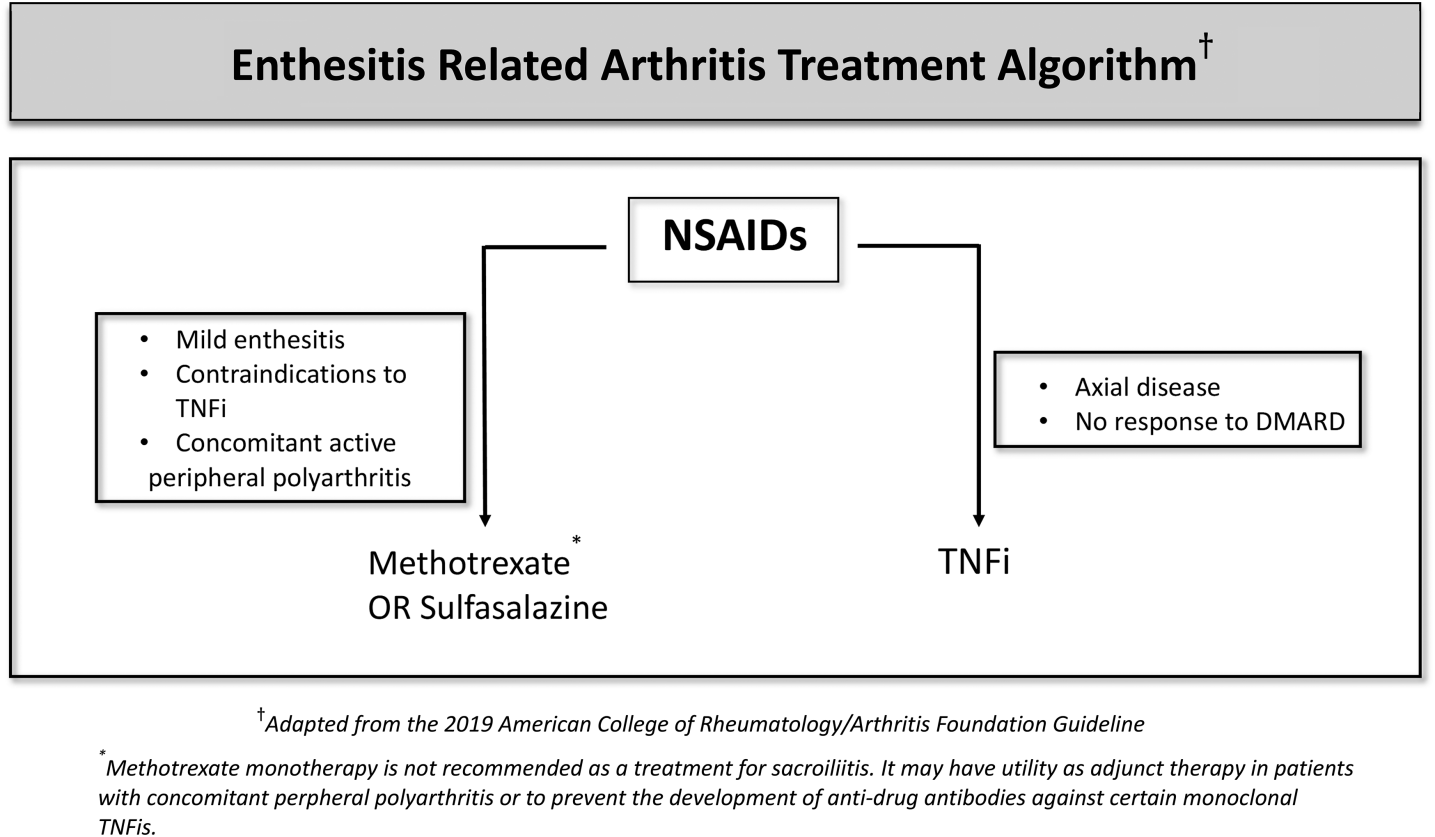

Recently anti-IL17 has become an alternative treatment in ERA as well. A total of 86 patients (52 ERA, 34 JSpA 34 patients; median age, 14 years) were enrolled for an open-label secukinumab trial in the first treatment period. In the second period, responders received secukinumab or placebo. Secukinumab demonstrated a significantly longer time to disease flare and a consistent safety profile similar to adults (170). Moreover, there are ongoing clinical trials for ixekizumab (NCT04527380).

Bridging therapy with systemic glucocorticoids might be used during the initiation or escalation of therapy. Intraarticular glucocorticoid injections of the sacroiliac joints as an adjunct therapy are conditionally recommended (165). Physiotherapy is also a crucial element in the treatment process thus should be offered to all SpA and JIA patients.

Another important aspect of the treatment is to monitor the side-effects of the drugs. For NSAIDs, gastrointestinal problems may arise thus proton pump inhibitors might also be prescribed. On the other hand, adequate fluid intake is essential to circumvent renal injury (171). Anti-TNF drugs make patients prone to infections thus in countries where tuberculosis is still encountered, routine screening should be performed.

Disease activity has to be followed to evaluate the response to treatment. For JIA patients, Juvenile Arthritis Disease Activity Score (JADAS) and Bath Ankylosing Spondylitis Disease Activity Index (BASDAI) are screened whereas BASDAI and Ankylosing Spondylitis Disease Activity (ASDAS) are used for the assessment of therapy success in adult SpAs (172, 173). These are applied for childhood diseases with axial involvement as well. Finally, Weiss et al. have developed and validated the first disease activity assessment for JSpA through international input and consensus formation techniques: this new criterion was called the Juvenile Spondyloarthritis Disease Activity (JSpADA) Index. This outcome tool had a good performance in discriminating between subjects with active vs. inactive disease and responded well to changes in the disease activity (174).

For adults, many therapeutics have been used and published. The reader is referred to excellent reviews on the subject. The primary treatment for SpA is NSAIDs and TNFis (certolizumab, etanercept, infliximab, adalimumab, and golimumab). Of note, SpA patients have higher levels of TNF-α (101) and HLA-B27 positive patients have a better response rate to TNF therapy (175) that might be explained by higher TNF levels in these patients (176). Therefore having TNF levels above a certain threshold value may help to estimate a better response and analysis of TNF levels before treatment might be beneficial.

wIL17 inhibitors (secukinumab and ixekizumab) can also be used for patients. Anti-IL17 is not recommended in patients with IBD or recurrent uveitis. If the patient has tuberculosis or recurrent infections, sulfasalazine is preferred over secukinumab and ixekizumab. Tofacitinib (a JAK inhibitor) is a second-line option for patients with contraindications to TNFi or anti-IL17. Co-treatment with low-dose methotrexate is not generally recommended except with infliximab (177). Brodalumab (IL17RA), bimekizumab (dual inhibition of IL17A and IL17F), and upadacitinib (selective JAK1 inhibitor) demonstrated improvement in active axial SpA (178–180). Although IL23 inhibitors (tildrakizumab, risankizumab and guselkumab) are effective in the treatment of psoriatic arthritis (181), in phase 2 and phase 3 studies, the use of ustekinumab and risankizumab did not show any improvement on SpA disease activity (182, 183). Of note, discontinuation of these biologic disease-modifying drugs (DMARDs) is not recommended due to the risk of flare (177) and all these biological DMARDs may be studied in adolescent patients as well.

## Conclusion

As summarized above, studies ongoing for more than 4 decades have led to the discovery of many risk factors for SpA development. Among these factors HLA-B27 seems to be the spearhead helping us to better understand the etiology of the disease. HLA-B27 driven mechanisms are thought to involve UPR activation and switching on the IL23/IL17 axis.

The fact that only a part of HLA-B27 positive people develop SpA indicates that there are additional factors contributing to disease pathogenesis. Although the threshold effect for HLA-B27 might be a possible explanation for this observation, it is most likely that further investigation of factors other than HLA-B27 is required that will also pave the way for the development of alternative therapies. The current treatment regimen involves NSAIDs, TNF inhibitors and possibly DMARDs. However, only some patients respond to the treatment which in turn causes a significant decrease in the non-responders' quality of life. Therapies targeting UPR and IL23/IL17 axis have recently gained attention but clinical trials are needed for further validation.
