# Supplementary material for: Spondyloarthritides: Theories and beyond
Source: Front Pediatr. 2022 Dec 23;10:1074239. doi: 10.3389/fped.2022.1074239 (PMC9816396; doi:10.3389/fped.2022.1074239)
Supplement: Supplementary file 1 [file Table1.docx]

| **ASAS classification criteria for peripheral spondyloarthritis** | **ASAS classification criteria for axial spondyloarthritis** | **ILAR classification criteria for enthesitis-related arthritis** |
| --- | --- | --- |
| Peripheral arthritis and/or enthesitis and/or dactylitis  PLUS  ≥1 SpA feature   - Uveitis - Psoriasis - Crohn‘s/colitis - Preceding infection - HLA-B27 - Sacroiliitis on imaging   OR  ≥2 other SpA features   - Arthritis - Enthesitis - Dactylitis - Inflammatory back pain (ever) - Family history of SpA | In patients with ≥3 months back pain and age <45 years  Sacroiliitis on imaging plus ≥1 SpA feature  OR  HLA-B27 plus ≥2 other SpA features  SpA features  • Inflammatory back pain  • Arthritis  • Enthesitis  • Uveitis  • Dactylitis  • Psoriasis  • Crohn‘s/colitis  • Good response to NSAIDs  • Family history of SpA  • HLA-B27  • Elevated CRP  Sacroiliitis on imaging  • Active (acute) inflammation on MRI highly suggestive of sacroiliitis associated with SpA  • Definite radiographic sacroiliitis according to modified New York criteria | Inclusion criteria  Arthritis and enthesitis  OR  Arthritis or enthesitis plus two of the following:   - Sacroiliac joint tenderness and/or inflammatory back pain - Presence of HLA-B27 - >6 years old boys - Acute anterior uveitis - Family history in at least one first degree relative of HLA-B27 associated disease (ankylosing spondylitis, ERA, sacroiliitis with IBD, reactive arthritis or acute anterior uveitis)   Exclusion criteria   - Psoriasis or a history of psoriasis in the patient or a first degree relative - Presence of IgM RF on at least two occasions at least 3 months apart - Presence of systemic arthritis |
